# Improving of bowel cleansing effect for polyethylene glycol with ascorbic acid using simethicone: A randomized controlled trial

**DOI:** 10.1097/MD.0000000000004163

**Published:** 2016-07-18

**Authors:** In Kyung Yoo, Yoon Tae Jeen, Seung Hun Kang, Jae Hyung Lee, Seung Han Kim, Jae Min Lee, Hyuk Soon Choi, Eun Sun Kim, Bora Keum, Hoon Jai Chun, Hong Sik Lee, Chang Duck Kim

**Affiliations:** Division of Gastroenterology and Hepatology, Department of Internal Medicine, Institute of Digestive Disease and Nutrition, Korea University College of Medicine, Seoul, Republic of Korea.

**Keywords:** ascorbic acid, bowel preparation, colonoscopy, polyethylene glycol, simethicone

## Abstract

**Background and Aim:**

Low-volume polyethylene glycol with ascorbic acid (PEG-Asc) use is reported to be as safe and effective as traditional 4-L polyethylene glycol use. However, PEG-Asc produces bubbles, which cause problems during colonoscopy. Data on the effects of using antifoaming agents such as simethicone with PEG-Asc are lacking. The aim of this CONSORT-prospective, randomized, observer-blinded, controlled trial is to compare the quality of bowel preparation and compliance between PEG-Asc users and PEG-Asc plus simethicone users.

**Methods:**

Adult outpatients aged 18 to 80 years undergoing colonoscopy were recruited to the study. Two hundred sixty patients were randomly assigned to 1 of 2 treatment arms, PEG-Asc or PEG-Asc plus simethicone. The primary outcome measure was the bowel cleansing quality using Boston bowel preparation scale and bubble scores. The secondary outcome measures were patient tolerability and doctor tolerability.

**Results:**

The simethicone group showed superior cleansing results (6–9 Boston scale scores: 99% vs. 84%, <5% bubble scores: 96% vs. 49%, *P* < 0.001) and fewer gastrointestinal symptoms (abdominal fullness: 24% vs. 55%, colicky pain: 5% vs. 24%, *P* < 0.001) than the non-simethicone group. Moreover, endoscopist fatigue during colonoscopy was lower in the simethicone group than in the non-simethicone group (1.31 ± 0.75 vs. 2.97 ± 2.14, *P* < 0.001).

**Conclusion:**

PEG-Asc plus simethicone use was more effective and associated with better patient and endoscopist tolerance than PEG-Asc use. Therefore, this combination is recommended as one of the promising methods for bowel preparation before colonoscopy.

## 1 Introduction

Optimal bowel preparation is essential for the efficacy and safety of colonoscopy. Mucosal visualization during colonoscopy is often limited by residual stool, bubbles, bile, intraluminal fluid, and debris, which increase the risk of missing flat adenomas or other small lesions.^[1,2]^ It is difficult for endoscopists to evaluate the mucosa by using images resulting from inadequate preparation, leading to decreased diagnostic accuracy, prolonged endoscopy duration, and decreased patient tolerance. Therefore, intestinal preparation is necessary to remove residual materials before endoscopy.

The most commonly used preparation regimens are based on polyethylene glycol (PEG). However, existing PEG-based preparations require the use of large volumes of product, which reduces patient tolerance and compliance, potentially resulting in inadequate cleansing. Low-volume hyperosmolar preparations have recently been developed. These preparations improve patient tolerance by reducing solution volumes and improving solution taste while offering cleansing effects similar to those of the standard large-volume preparations. Although low-volume bowel preparations have been shown to have superior bowel cleansing effects, it is still necessary to improve the overall cleansing efficacy and acceptability of bowel preparations for the visualization of colonic mucosa.

A low-volume PEG solution containing ascorbic acid (PEG-Asc) (CoolPrep; TaeJoon Pharmaceuticals, Seoul, Republic of Korea) is commonly used for bowel preparation in Republic of Korea. An excessive dose of ascorbic acid cannot be absorbed and functions as an osmotic laxative. It thereby reduces the necessary effective volume of the colon cleansing solution to 2 L.^[3]^ However, practitioners have noted an increased incidence of bubble formation with the use of this preparation method.^[4]^ The potential value of defoaming agents in mucosal toileting has long been recognized.^[5,6]^ However, there are few data regarding the effects of simethicone preparations on mucosal visibility during colonoscopy.^[7]^ Most studies have evaluated the effectiveness of adding simethicone to capsule endoscopy and gastroscopy preparation regimens.^[8]^ To our knowledge, no previous study has assessed the effects of colon preparation with simethicone in patients.

This study aimed to compare the quality of bowel preparation and compliance between patients receiving PEG-Asc and those receiving PEG-Asc plus simethicone. The effectiveness of using simethicone as an antifoaming agent to improve bowel cleansing for colonoscopy was evaluated using the bowel preparation scale and bubble scores, and patient and endoscopist compliance was assessed using a questionnaire.

## 2 Methods

### 2.1 Study design

This prospective, randomized, observer-blinded study was conducted in Korea University Hospital from July to September 2014. Two hundred sixty outpatients were included and assigned to 1 of 2 treatment groups: PEG-Asc and PEG-Asc plus simethicone. The study design complied with internationally recognized guidelines for clinical studies (ClinicalTrials.gov: NCT02548403). All patients provided written informed consent. This study was approved by the Institutional Review Board of Korea University Hospital (ED14306).

### 2.2 Patients

Adult outpatients aged 18 to 80 years undergoing colonoscopy were recruited to the study. Patients were considered ineligible for participation in the presence of any of the following: heart failure (New York Heart Association class III or IV), chronic kidney disease, untreated or uncontrolled hypertension, severe constipation as defined by Rome III criteria,^[9]^ major colonic resection, gastrointestinal obstruction, or significant gastroparesis.

### 2.3 Randomization

Patients were randomly assigned to 1 of 2 fixed-dose treatment arms, PEG-Asc or PEG-Asc plus simethicone. Randomization numbers were allocated sequentially to the participants by using a computer-generated randomization list with a block of 4. The endoscopists did not participate in the randomization process. Patients were randomly allocated to receive 1 of the 2 bowel preparations on a 1:1 basis, PEG-Asc or PEG-Asc plus simethicone. Patients were training so as not to consult with staff member about their preparation kit.

### 2.4 Preparation methods

The study preparation was a PEG-Asc plus simethicone. On the day before colonoscopy, the first 1 L of PEG-Asc solution was given at 7 to 8 pm, and the remaining 1 L of solution was given 5 hours before colonoscopy. After each 1 L of solution, patients were instructed to drink 500 mL of additional clear fluid. Two packs (200 mg/10 mL each) of simethicone (400 mg) were mixed with the last 500 mL of additional clear fluid.

As a control, PEG-Asc (100 g of PEG 3350 plus ascorbic acid and electrolytes) with 1 L additional clear fluid was used. On the day before colonoscopy, the first 1 L of PEG-Asc solution was given at 7 to 8 pm, and the remaining 1 L of solution was given 5 hours before colonoscopy. After each 1 L of solution, patients were instructed to drink 500 mL of additional clear fluid.

Patients had to finish a liquid supper at least 1 hour before taking the preparation solution. Clear fluids were permitted until midnight. Then, patients underwent colonoscopy between 8:00 am and 1:00 pm.

The participants received instructions on how to take the preparation products. It was unaccepted for patients to eat solid food including high-fiber vegetables, fruit seeds, or mixed grains starting from 3 days before the colonoscopy.

### 2.5 Evaluation of bowel preparations

#### 2.5.1 Assessment of bowel cleansing efficacy

Overall quality of colon preparation was assessed using the Boston bowel preparation scale (BBPS) and bubble scores. The BBPS^[10]^ is a bowel cleansing rating scale using 4-point scale (0–3) applied to 3 individual broad segments of the colon (the right, transverse, and rectosigmoid colon). The total bowel cleansing scores may range from 0 (unprepared colon) to 9 (perfectly clean colon). In this study, total BBPS scores ≥6 were considered as successful bowel preparation.

We also measured the bubble score, which is categorized by the amount of foam and bubbles related to colonic mucosal visualization.^[4,11]^ The scores were assigned in accordance with the degree of obscuration by bubbles, bile, or debris as follows: 0 (<50% of mucosa seen, severe obscuration), 1 (50%–75% of mucosa seen, moderate obscuration), 2 (80%–95% of mucosa seen, mild obscuration), and 3 (>95% of mucosa seen, no obscuration).

Experienced endoscopists made every effort to reduce interobserver variability when rating the bowel preparation quality. Before study initiation, these experienced colonoscopists performed calibration exercises about scoring systems such as BBPS or bubble score to reach a satisfactory level of concordance.

#### 2.5.2 Assessment of safety

Physical examination and vital sign assessment were performed at the time of patient enrollment and on the day of colonoscopy. Patients were asked to complete a questionnaire before the colonoscopy to evaluate adverse events related to the preparations. Newly developed preparation-related symptoms or exacerbations of preexisting symptoms (except those included in the evaluation of gastrointestinal tolerability) were assessed.

#### 2.5.3 Assessment of patient tolerability

Patients were given the standardized questionnaires before the colonoscopy to evaluate the compliance. They were asked about preparation-associated symptoms such as abdominal fullness, colicky pain, nausea, vomiting, sleep disturbance, and general discomfort. A 5-point scale^[12]^ (from 1 [=excellent] to 5 [bad]) was used to score the taste of the bowel cleansing agents.

#### 2.5.4 Assessment of doctor tolerability

Endoscopist fatigue during colonoscopy was scored using a visual analog scale, ranging from 1 to 10, where 1 and 10 represented “strongly disagree” and “strongly agree,” respectively. To investigate the factors associated with fatigue, responses to a questionnaire regarding water shooting counts were assessed. The questionnaires were completed by the participating endoscopists immediately after each colonoscopy.

### 2.6 Statistical analysis

The sample size calculation was designed to detect differences in treatment success, using α of 0.05, a power of 0.80, and 5% dropout rate. Because there are no published data regarding the addition of simethicone to the PEG-Asc preparation, our sample size was calculated based on a previous study performed in Korea University Hospital with 30 patients in each group. The adequate rate efficacies based on the BBPS were an estimated 85% and 95% for PEG-Asc and PEG-Asc plus simethicone, respectively. A sample size of 133 patients per group was required, which was increased to approximately 270 total patients. A 2-sided *t* test was used to compare the means of continuous variables, which were expressed as mean ± standard deviation. Chi-square tests were used to compare the rates of categorical variables, which were expressed as counts and percentages. In addition, fatigue levels associated with various clinical parameters were analyzed using the Spearman rank correlation test.

For all analyses, the criterion for significance was *P* < 0.05. Statistical analyses were performed by using SPSS Statistics (version 20.0, IBM Corp, Armonk, NY).

## 3 Results

### 3.1 Baseline characteristics

Of the 270 patients who underwent randomization, 10 were excluded from analysis (did not meet inclusion criteria in 7, withdrawal of informed consent in 3 cases). Thus, 260 patients were assessed for eligibility and assigned to 1 of 2 groups. The baseline characteristics of the patients were similar for the 2 groups (Table 1). As shown in Table 2, there were no significant differences in complete understanding of pretest, food restrictions, nothing-by-mouth times and colonoscopic variables such as cecal intubation times, and adenomatous polyp detection rates between the groups. However, the withdrawal time was significantly shorter in the PEG-Asc plus simethicone group than in the PEG-Asc group (13.35 ± 7.86 vs. 17.29 ± 13.17 minutes, *P* < 0.05).

**Table 1:**
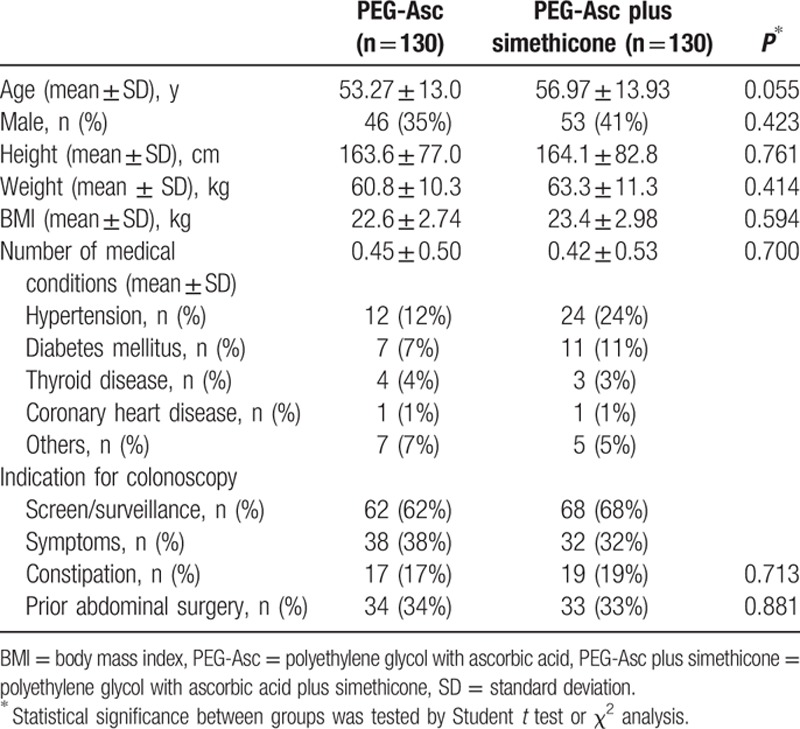
Baseline characteristics.

**Table 2:**
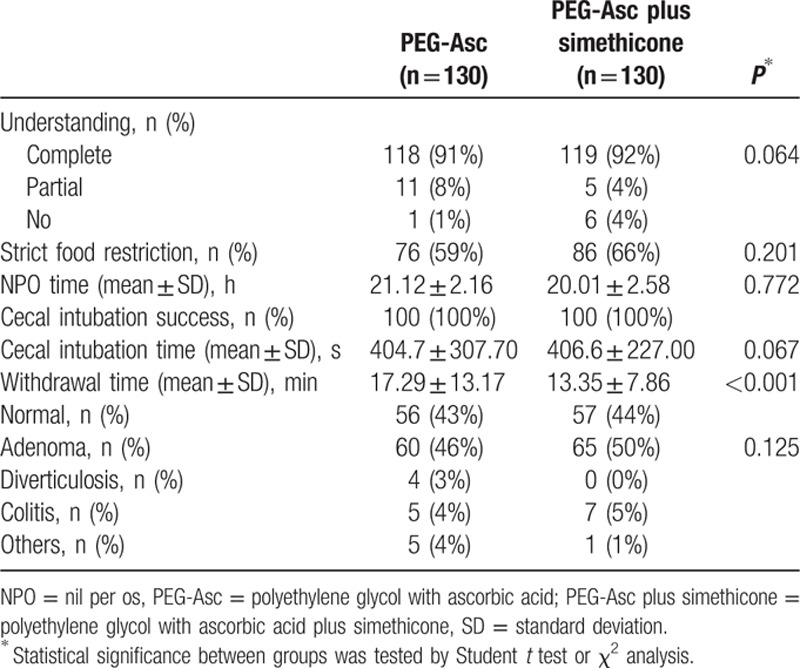
Bowel preparation characteristics and colonoscopy results.

### 3.2 Efficacy of bowel cleansing

#### 3.2.1 BBPS

**Figure 1:**
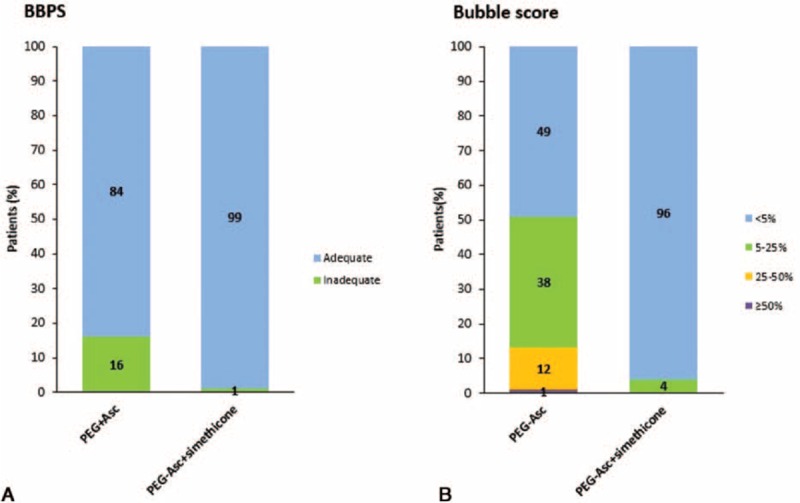
Comparison of bowel cleansing efficacy: (A) using the Boston bowel preparation scale; (B) using bubble scores. PEG-Asc = polyethylene glycol with ascorbic acid, PEG-Asc + simethicone = polyethylene glycol with ascorbic acid plus simethicone.

Colonoscopy cleansing data were obtained from all 260 patients. Table 3 shows the bowel cleansing quality scores based on the BBPS. Both methods showed successful cleansing effects, with scores of 6.70 and 7.82 in the PEG-Asc and PEG-Asc plus simethicone groups, respectively; the difference in the mean total scores was borderline significant (*P* = 0.058). The mean BBPS scores at the right and middle colon segments were almost the same: 2.22 and 2.26 for the PEG-Asc group, and 2.43 and 2.61 for the PEG-Asc plus simethicone group, respectively. However, the mean BBPS scores for the rectosigmoid and total colon segments were significantly higher in the PEG-Asc plus simethicone group than in the PEG-Asc group. Furthermore, successful bowel cleansing was observed in more patients in the PEG-Asc plus simethicone group (total BBPS score ≥6) than in the PEG-Asc group (99% [95% confidence interval (CI) 96–100] vs. 84% [95% CI 77–90], *P* < 0.05; Fig. 1A).

**Table 3:**
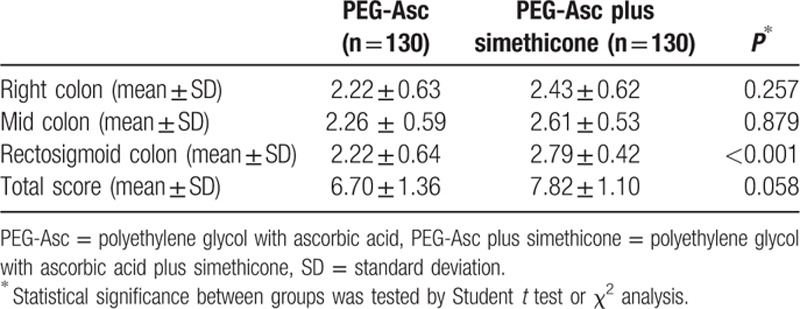
The efficacy of bowel cleansing (Boston scale).

#### 3.2.2 Bubble scores

**Figure 2:**
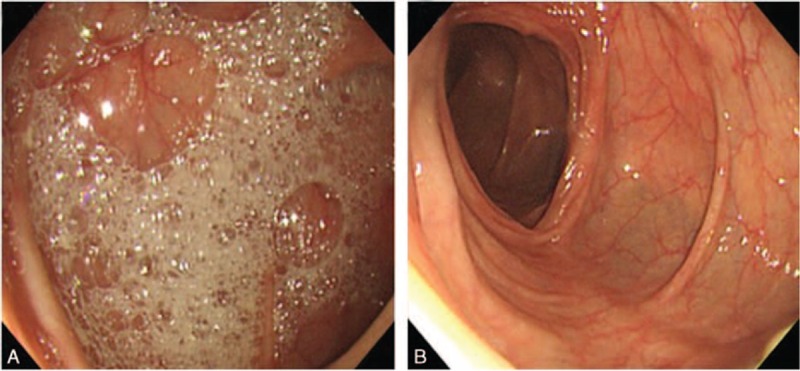
Comparison of the representative colonoscopy images from the 2 treatment groups: (A) severe obscuration caused by intraluminal gas bubbles with PEG-Asc use; (B) excellent visibility with no intraluminal gas bubbles with PEG-Asc plus simethicone use. PEG-Asc = polyethylene glycol with ascorbic acid, PEG-Asc plus simethicone = polyethylene glycol with ascorbic acid plus simethicone.

Comparisons of the bowel cleansing quality in terms of the bubble scores are shown in Fig. 1B. The percentage of patients showing <5% bubbles during colonoscopy was significantly higher in the PEG-Asc plus simethicone group than in the PEG-Asc group (96% [95% CI: 93–100] vs. 49% [95% CI: 41–58], *P* < 0.05). Representative colonoscopy images from each group are shown in Fig. 2, in which a marked decrease in gas bubbles is observed in the PEG-Asc plus simethicone group compared with that in the PEG-Asc group.

### 3.3 Patient tolerance

The incidences of symptoms associated with bowel preparation, including abdominal fullness, colicky pain, nausea, vomiting, sleep disturbance, and general discomfort, in the 2 groups are shown in Table 4. Analysis of specific symptoms revealed that instances of abdominal fullness and colicky pain were significantly lower in the PEG-Asc plus simethicone group than in the PEG-Asc group (24% [95% CI: 16–31] vs. 55% [95% CI: 46–63]; 5% [95% CI: 2–10] vs. 24% [95% CI: 16–31]; *P* < 0.001, respectively). However, there were no significant differences in other tolerability factors including taste between the groups.

**Table 4:**
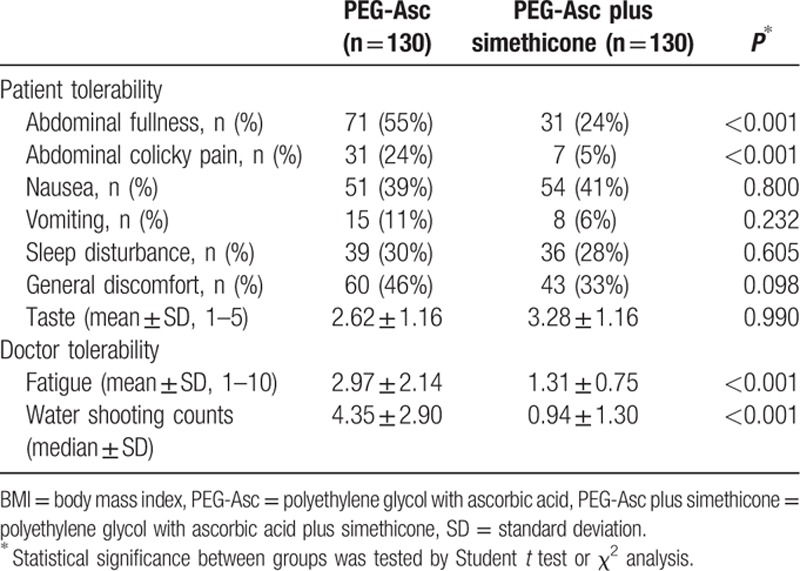
Patient and endoscopist tolerability for bowel preparation.

### 3.4 Endoscopist tolerance

From the perspective of the endoscopists, use of PEG-Asc plus simethicone resulted in fewer bubbles that disturbed the lens. Analysis of fatigue rated from 1 (no fatigue) to 10 (greatly fatigued) revealed that endoscopists experienced less fatigue when evaluating PEG-Asc plus simethicone group patients than when evaluating PEG-Asc group patients (1.31 ± 0.75 vs. 2.97 ± 2.14, *P* < 0.001). The counts of water shooting for cleaning the lens were also significantly lower in the PEG-Asc plus simethicone group, as shown in Table 4 (0.94 ± 1.30 vs. 4.35 ± 2.90, *P* < 0.001). The endoscopist fatigue scores were analyzed in terms of various clinical parameters such as age, sex, body mass index, number of medical conditions, constipation, withdrawal time, and water shooting counts. As shown in Table 5, the fatigue scores were significantly correlated only with the water shooting counts (Spearman rank correlation coefficient, r = 0.689, *P* < 0.001).

**Table 5:**
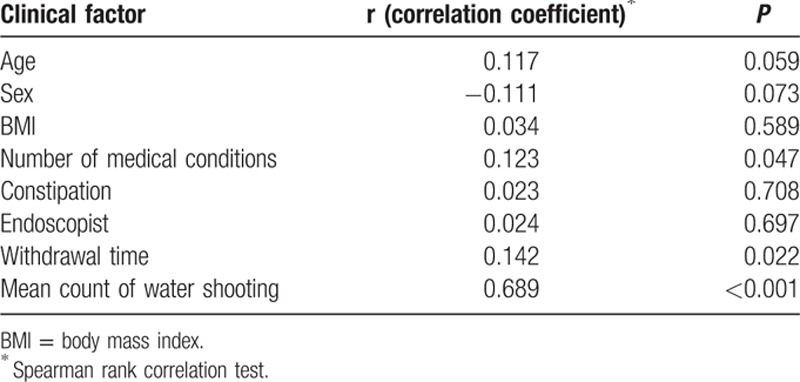
Relationship between doctor's fatigue level and clinical factors.

### 3.5 Patient safety

No serious adverse events occurred in either group. Moreover, no noteworthy vital signs were observed before or after colonoscopy. Only minor adverse events of preparation-related symptoms (i.e., abdominal fullness, colicky pain, nausea, vomiting, general discomfort, and sleep disturbance) evaluated as factors related to patient tolerance were recorded by the questionnaires.

## 4 Discussion

In this study, the effects of a combined regimen of PEG-Asc plus simethicone were compared with those of a regimen of PEG-Asc alone. This study aimed to determine the effectiveness of simethicone in low-volume bowel preparation before colonoscopy. This combined simethicone regimen was observed to be feasible and effective for colonoscopy preparation. Simethicone use significantly reduced the formation of colonic air bubbles and foam and reduced the incidence of abdominal fullness and colicky pain (24% vs. 55% and 5% vs. 24%, simethicone group vs. non-simethicone group, respectively; *P* < 0.001). Another noteworthy finding in this study was the decrease in the number of water shooting counts during colonoscopy in the simethicone group, which resulted in reduced fatigue. These results suggest that simethicone use is associated with patient and physician satisfaction.

Simethicone is a detergent mixture of dimethylpolysiloxane and silica gel. It is physiologically inactive and nontoxic. It can be taken orally and cannot be absorbed by the gastrointestinal system.^[13]^ It disrupts air bubbles by reducing their surface tension. Simethicone is generally used to treat patients with symptoms caused by excess gas in the intestinal tract. However, to date, there have been no reliable reports on its routine use for colonoscopy preparation. According to our data, colonic bubbles occurring during colonoscopies can be almost entirely removed by adding simethicone to the PEG-Asc preparation. This result may have practical implications: simethicone is an economical, easy-to-take, antifoaming agent with proven safety.^[14]^

Our study has several strengths and important differences from previous studies. First, to our knowledge, this is the first study to evaluate the effects of low-volume PEG-Asc combined with simethicone. Several studies have compared colon preparation between patients receiving purgatives plus simethicone and those receiving only purgatives.^[15–18]^ PEG or sodium phosphate solutions were usually used for bowel preparation in these patients. However, no previous study has assessed the effects of simethicone combined with low-volume PEG-Asc on endoscopic visibility and diagnostic yield. We believe that our results are associated with the types of drug preparations. According to our clinical experience, PEG-Asc use increases the incidence of bubble formation. Therefore, we believed that adding simethicone to PEG-Asc solution would result in improved conditions. In the present study, bubble scoring was performed to assess the differences between the treatment groups. This scoring system allowed a qualitative assessment of the effects of using PEG-Asc plus simethicone.

Second, the appropriate timing of simethicone administration may be important. In this study, simethicone was administered with the last free water consumed during the preparation. However, there is currently no consensus regarding the appropriate timing of its administration. This study followed the published methods describing the use of simethicone before capsule endoscopy.^[19–22]^ Furthermore, the time lag caused by colonic emptying should be considered when drug administration is timed. Although these findings must be confirmed in future trials, the cleansing effects observed in our study suggest the usefulness of this method. Appropriate preparation methods should remove bubbles, have minimal side effects, be tolerated by patients, and be applicable to most patients in a variety of conditions.

Third, this is the first prospective study to evaluate the relationship between endoscopist fatigue and the preparation method used and to examine the effectiveness of simethicone in bowel preparation. Fatigue is a common complaint among endoscopists and has been reported to increase medical or cognitive errors.^[23,24]^ The preparation method used in the present study has been shown to improve endoscopist experience. The presence of bubbles may increase operator fatigue and interfere with endoscopic visualization. Bubbles and foam in the intestinal lumen often hamper the endoscopist's view, leading to multiple aspirations of the adherent foam and intraprocedural lavages, thus increasing endoscopist fatigue. The addition of simethicone is well tolerated by the endoscopists and may reduce the overall procedure time, although the withdrawal time was not significantly correlated with fatigue in our study.

In this study, bowel preparation with simethicone allowed more effective bowel cleansing during colonoscopy than bowel preparation without simethicone. A higher number of simethicone group patients reported “adequate” bowel preparation quality than non-simethicone group patients (6–9 Boston scale score: 99% vs. 84%, <5% bubble score: 96% vs. 49%, *P* < 0.005). The presence of bubbles may lower the detection rate of polyps and colorectal cancer during colonoscopy.

The major limitation of our study is that this randomized controlled trial was conducted at a single center and included only outpatients without significant medical problems. Multicenter randomized trials with an unselected group of patients are required for further confirmation of the results. Moreover, we did not thoroughly evaluate safety issues or perform hematological or biochemical analysis of patient blood in this study. However, clinically significant complications did not newly develop or aggravate. Fatigue grading systems have not been standardized, which causes difficulties in analyzing the results and applying them to routine clinical practice. We cannot ascertain how colon preparation affects endoscopist fatigue in different medical situations. However, these limitations applied to both the groups.

In conclusion, this study shows that simethicone use may reduce intraluminal bubbles in patients receiving PEG-Asc preparations. Simethicone use improves bowel mucosal visualization and tolerability of bowel preparation for colonoscopy. An effective low-volume bowel preparation method associated with high patient tolerance and improved treatment adherence has been recommended. However, there is no consensus regarding the best bowel preparation method for colonoscopy. We believe that the use of PEG-Asc plus simethicone is a promising bowel preparation method for colonoscopy.

## Supplementary Material

SUPPLEMENTARY MATERIAL
