# Supplementary material for: Improving of bowel cleansing effect for polyethylene glycol with ascorbic acid using simethicone: A randomized controlled trial
Source: Medicine (Baltimore). 2016 Jul 18;95(28):e4163. doi: 10.1097/MD.0000000000004163 (PMC4956803; doi:10.1097/MD.0000000000004163)
Supplement: SUPPLEMENTARY MATERIAL [file medi-95-e4163-s001.docx]

Table S1

| PPM | CDAF | NDAF | P-value |
| --- | --- | --- | --- |
| (n=697) | (n=92) | (n=605) |  |
| Age at procedure (years) | 67.1±13.3 | 65.1±13.1 | 0.16 |
| Male | 32 (34.8) | 274 (45.3) | 0.0 |
| Atrial lead | 45 (48.9) | 459 (75.9) | <0.001 |
| **Medical History** |  |  |  |
| Hypertension | 57 (62.0) | 351 (58.0) | 0.47 |
| Diabetes | 17 (18.5) | 138 (22.8) | 0.35 |
| Renal insufficiency | 3 (3.3) | 32 (5.3) | 0.40 |
| Previous coronary artery disease | 14 (15.2) | 104 (17.2) | 0.63 |
| Previous peripheral artery disease | 2 (2.2) | 61 (10.1) | 0.01 |
| Prior heart failure | 10 (10.9) | 38 (6.3) | 0.10 |
| Prior stroke | 12 (13.0) | 53 (8.8) | 0.18 |
| Prior sinus node dysfunction | 55 (59.8) | 179 (29.6) | <0.001 |
| Prior symptomatic AV block | 49 (53.3) | 427 (70.6) | <0.001 |
| CHA_2_DS_2_-VASc at procedure date | 3.0 (1.3-4.0) | 2.0 (1.0-3.0) | 0.18 |
| **Echocardiographic profile** |  |  |  |
| Mitral valve disease | 5 (5.4) | 14 (2.3) | 0.08 |
| Aortic valve disease | 1 (1.1) | 11 (1.8) | 0.61 |
| Ejection fraction < 40% | 2 (2.2) | 25 (4.1) | 0.36 |
| LVEDd (mm) | 50.0 (42.3-54.0) | 51.0 (48.0-54.0) | 0.06 |
| LVESd (mm) | 29.5 (27.0-32.8) | 30.0 (27.0-33.0) | 0.17 |
| Left atrium volume index ≥38.5 (ml/m²) | 78 (84.8) | 426 (70.4) | 0.004 |
| **Medication at procedure** |  |  |  |
| Beta-blocker | 11 (12.0) | 57 (9.4) | 0.44 |
| ACE inhibitor or ARB | 34 (37.0) | 201 (33.2) | 0.48 |
| Statin | 10 (10.9) | 106 (17.5) | 0.11 |
| Anti-platelet therapy | 32 (34.8) | 155 (25.6) | 0.07 |
| Anti-coagulation therapy | 4 (4.3) | 17 (2.8) | 0.42 |
| Diuretics | 16 (17.4) | 117 (19.3) | 0.65 |
| Aldosterone antagonist | 2 (2.2) | 20 (3.3) | 0.56 |

| ICD or CRT | CDAF | NDAF | P-value |
| --- | --- | --- | --- |
| (n=183) | (n=30) | (n=153) |  |
| Age at procedure (years) | 55.1 ± 13.1 | 52.0 ± 15.0 | 0.29 |
| Male | 6 (20.0) | 32 (20.9) | 0.91 |
| Atrial lead | 6 (3.3) | 19 (10.4) | 0.25 |
| **Medical History** |  |  |  |
| Hypertension | 8 (26.7) | 29 (19.0) | 0.33 |
| Diabetes | 10 (33.3) | 52 (34.0) | 0.94 |
| Renal insufficiency | 2 (6.7) | 10 (6.5) | 0.97 |
| Previous coronary artery disease | 6 (20.0) | 27 (17.6) | 0.75 |
| Previous peripheral artery disease | 10 (33.3) | 21 (13.7) | 0.009 |
| Prior heart failure | 12 (40.0) | 55 (35.9) | 0.67 |
| Prior stroke | 1 (3.3) | 4 (2.6) | 0.82 |
| Prior sinus node dysfunction | 0 (-) | 0 (-) | . |
| Prior symptomatic AV block | 1 (3.3) | 0 (-) | 0.16 |
| CHA_2_DS_2_-VASc at procedure date | 2.0 (2.0-3.0) | 2.0 (1.0-3.0) | 0.18 |
| **Echocardiographic profile** |  |  |  |
| Mitral valve disease | 0 (-) | 0 (-) | . |
| Aortic valve disease | 0 (-) | 0 (-) | . |
| Ejection fraction < 40% | 14 (46.7) | 74 (48.4) | 0.86 |
| LVEDd (mm) | 58.0 (49.7-71.0) | 58.0 (49.0-67.0) | 0.50 |
| LVESd (mm) | 43.5 (28.7-61.2) | 42.0 (30.0-55.0) | 0.70 |
| Left atrium volume index ≥38.5 (ml/m²) | 25 (83.3) | 87 (56.9) | 0.007 |
| **Medication at procedure** |  |  |  |
| Beta-blocker | 19 (63.3) | 105 (68.6) | 0.57 |
| ACE inhibitor or ARB | 17 (56.7) | 74 (18.4) | 0.40 |
| Statin | 8 (26.7) | 49 (32.0) | 0.56 |
| Anti-platelet therapy | 9 (30.0) | 39 (25.5) | 0.60 |
| Anti-coagulation therapy | 3 (10.0) | 16 (10.5) | 0.94 |
| Diuretics | 10 (33.3) | 41 (26.8) | 0.46 |
| Aldosterone antagonist | 9 (30.0) | 32 (20.9) | 0.27 |

Table S2

| CIED | PPM | ICD or CRT | P-value |
| --- | --- | --- | --- |
| (n=880) | (n=697) | (n=183) |  |
| Age at procedure (years) | 65.3 ± 13.1 | 52.5 ± 14.7 | <0.001 |
| Male | 306 (43.9) | 38 (20.8) | 0.001 |
| Atrial lead | 504 (72.3) | 25 (13.7) | 0.001 |
| **Medical History** |  |  |  |
| Hypertension | 408 (58.5)) | 37 (20.2) | 0.001 |
| Diabetes | 155 (22.2) | 62 (33.9) | 0.01 |
| Renal insufficiency | 35 (5.0) | 12 (6.6) | 0.411 |
| Previous coronary artery disease | 118 (16.9) | 33 (18.0) | 0.725 |
| Previous peripheral artery disease | 63 (9.0) | 31 (16.9) | 0.002 |
| Prior heart failure | 48 (6.9) | 67 (36.6) | <0.001 |
| Prior stroke | 65 (9.3) | 5 (2.7) | 0.003 |
| Prior sinus node dysfunction | 234 (33.6) | 0 (-) | . |
| Prior symptomatic AV block | 476 (68.3) | 1 (0.5) | <0.001 |
| CHA_2_DS_2_-VASc at procedure date | 2.0 (1.0-4.0) | 2.0 (1.0-3.0) | 0.002 |
| **Echocardiographic profile** |  |  |  |
| Mitral valve disease | 19 (2.7) | 0 (-) | 0.024 |
| Aortic valve disease | 12 (1.7) | 0 (-) | 0.074 |
| Ejection fraction < 40% | 27 (3.9) | 88 (48.1) | <0.001 |
| LVEDd (mm) | 51.0 (48.0-54.0) | 58.0 (49.0-67.0) | <0.001 |
| LVESd (mm) | 30.0 (27.0-33.0) | 42.0 (30.0-55.0) | <0.001 |
| Left atrium volume index ≥38.5 (ml/m²) | 504 (72.3) | 112 (61.2) | 0.004 |
| **Medication at procedure** |  |  |  |
| Beta-blocker | 68 (9.8) | 124 (67.8) | <0.001 |
| ACE inhibitor or ARB | 235 (33.7) | 91 (49.7) | <0.001 |
| Statin | 116 (16.6) | 57 (31.1) | 0.001 |
| Anti-platelet therapy | 187 (26.8) | 48 (26.2) | 0.870 |
| Anti-coagulation therapy | 21 (3.0) | 19 (10.4) | <0.001 |
| Diuretics | 133 (19.1) | 51 (27.9) | 0.009 |
| Aldosterone antagonist | 22 (3.2) | 41 (22.4) | <0.001 |

Table S3
